# Toxin Profiles of Okadaic Acid Analogues and Other Lipophilic Toxins in *Dinophysis* from Japanese Coastal Waters

**DOI:** 10.3390/toxins10110457

**Published:** 2018-11-06

**Authors:** Hajime Uchida, Ryuichi Watanabe, Ryoji Matsushima, Hiroshi Oikawa, Satoshi Nagai, Takashi Kamiyama, Katsuhisa Baba, Akira Miyazono, Yuki Kosaka, Shinnosuke Kaga, Yukihiko Matsuyama, Toshiyuki Suzuki

**Affiliations:** 1 National Research Institute of Fisheries Science, Japan Fisheries Research and Education Agency, 2-12-4 Fukuura, Kanazawa-ku, Yokohama, Kanagawa 236-8648, Japan; huchida@affrc.go.jp (H.U.); rwatanabe@affrc.go.jp (R.W.); matsur@affrc.go.jp (R.M.); oikawah@affrc.go.jp (H.O.); snagai@affrc.go.jp (S.N.); 2 National Research Institute of Fisheries and Environment of Inland Sea, Japan Fisheries Research and Education Agency, 2-17-5, Maruishi, Hatsukaichi, Hiroshima 739-0452, Japan; kamiyama@affrc.go.jp; 3 Central Fisheries Research Institute, Fisheries Research Department, Hokkaido Research Organization, 238, Hamanakacho, Yoichi-cho, Yoichi-gun, Hokkaido 046-8555, Japan; baba-katuhisa@hro.or.jp; 4 Kushiro Fisheries Research Institute, Fisheries Research Department, Hokkaido Research Organization, 4-25, Nakahamacho, Kushiro-city, Hokkaido 085-0027, Japan; miyazono-akira@hro.or.jp; 5 Aomori Prefectural Industrial Technology Research Center, Fisheries Research Institute, Hiranai, Higashitsugarugun, Aomori 039-3381, Japan; yuuki_kosaka@aomori-itc.or.jp; 6 Iwate Fisheries Technology Center, 3-75-3 Hirata, Kamaishi, Iwate 026-0001, Japan; s-kaga@pref.iwate.jp; 7 Seikai National Fisheries Research Institute, Japan Fisheries Research and Education Agency, 1551-8, Taira-machi, Nagasaki-shi, Nagasaki 851-2213, Japan; yukihiko@affrc.go.jp

**Keywords:** *Dinophysis*, diarrhetic shellfish poisoning, marine toxins, pectenotoxin, okadaic acid, dinophysistoxin

## Abstract

The identification and quantification of okadaic acid (OA)/dinophysistoxin (DTX) analogues and pectenotoxins (PTXs) in *Dinophysis* samples collected from coastal locations around Japan were evaluated by liquid chromatography mass spectrometry. The species identified and analyzed included *Dinophysis fortii*, *D. acuminata*, *D. mitra* (*Phalacroma mitra*), *D. norvegica*, *D. infundibulus*, *D. tripos*, *D. caudata*, *D. rotundata* (*Phalacroma rotundatum*), and *D. rudgei*. The dominant toxin found in *D. acuminata* was PTX2 although some samples contained DTX1 as a minor toxin. *D. acuminata* specimens isolated from the southwestern regions (Takada and Hiroshima) showed characteristic toxin profiles, with only OA detected in samples collected from Takada. In contrast, both OA and DTX1, in addition to a larger proportion of PTX2, were detected in *D. acuminata* from Hiroshima. *D. fortii* showed a toxin profile dominated by PTX2 although this species had higher levels of DTX1 than *D. acuminata*. OA was detected as a minor toxin in some *D. fortii* samples collected from Yakumo, Noheji, and Hakata. PTX2 was also the dominant toxin found among other *Dinophysis* species analyzed, such as *D. norvegica*, *D. tripos*, and *D. caudata*, although some pooled picked cells of these species contained trace levels of OA or DTX1. The results obtained in this study re-confirm that cellular toxin content and profiles are different even among strains of the same species.

## 1. Introduction

**Figure 1:**
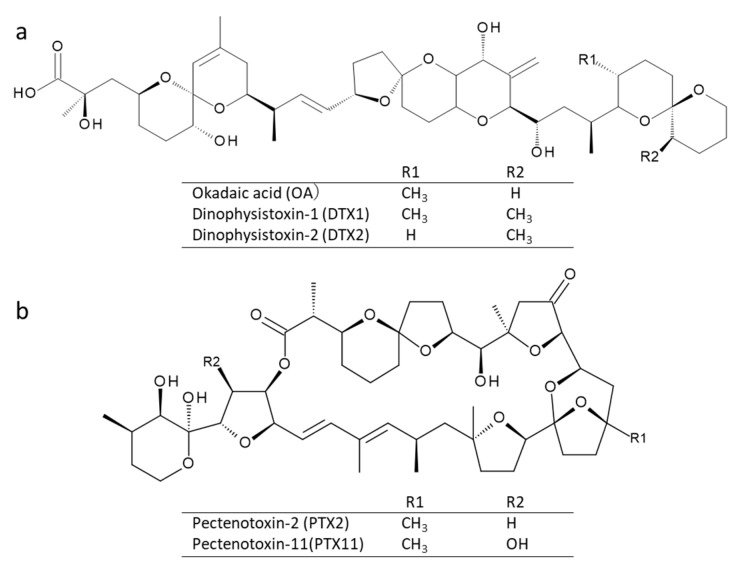
Chemical structure of okadaic acid (OA) and dinophysistoxin (DTX) and pectenotoxin (PTX) analogues found in *Dinophysis* species. (**a**) OA and DTX analogues. (**b**) PTX2 and PTX11.

The diarrhetic shellfish toxins (DSTs), okadaic acid (OA) and dinophysistoxins (DTXs), as well as pectenotoxins (PTXs) (Figure 1) [1], are produced by planktonic species of the genus, *Dinophysis* and benthic species of *Prorocentrum* [2]. Bivalves become contaminated with these marine toxins by feeding on toxic *Dinophysis* species. The regulation of DSTs recommended by Codex Alimentarius [3] is 160 ng OA equivalent/g in the edible part of bivalves. The regulation in the European Union (EU) is a total of 160 ng OA/DTX and PTXs/g in the edible part of bivalves [4]. The cellular toxin content and profiles of several *Dinophysis* species have been reported by analyzing field multispecific samples obtained by plankton net hauls, or monospecific cultures [5,6,7,8,9,10,11,12,13,14,15,16,17,18]. However, it remains important to update toxin content and profile information of *Dinophysis* species to improve the prediction of bivalve contamination. The cellular toxin content and profiles of *Dinophysis* species of pooled picked cells reported in previous studies was revised (Table 1) [19,20,21,22,23,24,25,26,27,28,29,30,31,32,33,34,35]. Analysis of individually picked cells was historically the only unambiguous way to ascribe a toxin profile and content information to a *Dinophysis* species, until 2006, when cultures of *D. acuminata* became available [36]. Because the cellular toxin content and profiles are different even among samples of the same species [36,37], it is necessary to clarify cellular toxin contents and profiles of *Dinophysis* spp. present in each bivalve monitoring area.

Historically, DST contamination of bivalves, and associated human poisoning cases, were restricted in the northeastern regions of Japan (Tohoku and Hokkaido area). Therefore, data on the toxin content and profiles of *Dinophysis* from these regions is essential for predicting bivalve contamination. Although *Dinophysis* occurs in the southwestern parts of Japan, no toxin information has been reported for *Dinophysis* species found there. It is interesting that DST positive cases in bivalves obtained with the previous DST official testing method (mouse bioassay) in the southwestern parts of Japan have hardly been reported.

Between 2006 and 2014, pooled picked cells of many *Dinophysis* species were generated from seawater samples taken from many locations around the Japanese coastline. DSTs and PTXs were extracted using a solid phase extraction method [6,19,23], and the extracts kept frozen until analysis. In this study, the presence of DSTs and PTXs in these samples was determined by liquid chromatography triple quadrupole tandem mass spectrometry (LC/MS/MS) [23] and liquid chromatography quadrupole mass spectrometry (LC/MS) [38].

## 2. Results

### 2.1. Dinophysis acuminata

**Figure 2:**
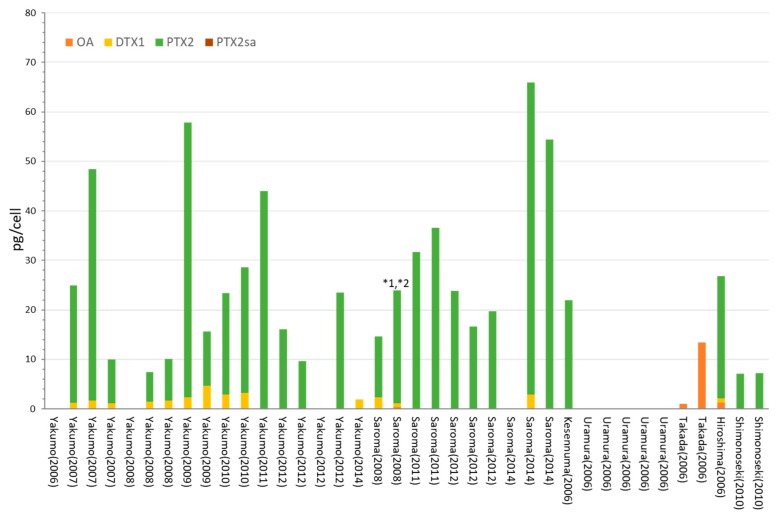
The toxin contents and profiles of *D. acuminata* in Japan. * 1 Trace levels of OA were detected. * 2 Trace levels of DTX1 were detected. The toxin contents, profiles, analyzed cell numbers, and detection limits for negative values are also given in Table S1.

The toxin content and profiles of *D. acuminata* obtained in this study are shown in Figure 2 and Table S1. The dominant toxin in *D. acuminata* samples from Yakumo, Saroma, and Shimonoseki was PTX2, and DTX1 was also observed at lower levels in some samples from Yakumo and Saroma. The DTX1 content (4.7 pg/cell) found in *D. acuminata* sample collected in Saroma was greater than the highest value of (0.7 pg/cell) reported in previous studies (Table 1) [23]. The toxin profile and contents found from *D. acuminata* in Yakumo were close to those obtained in a previous study for *D. acuminata* in the same area [23]. It is interesting that *D. acuminata* collected in Uramura did not produce any of the monitored toxins. *D. acuminata* collected in Takada and Hiroshima showed characteristic toxin profiles, with OA exclusively detected in *D. acuminata* collected in Takada, whereas both OA and DTX1, in addition to a higher proportions of PTX2, were detected in *D. acuminata* from Hiroshima.

### 2.2. Dinophysis fortii

**Figure 3:**
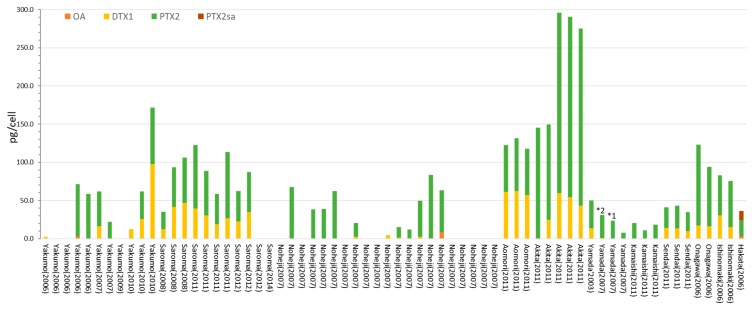
The toxin contents and profiles of *D. fortii* in Japan. * 1 Trace levels of OA were detected. * 2 Trace levels of DTX1 were detected. The toxin contents, the profiles, analyzed cell numbers, and detection limits for negative values are also given in Table S1.

The toxin content and profiles of *D. fortii* obtained in the present study are shown in Figure 3 and Table S1. Although the dominant toxin observed in *D. fortii* samples was PTX2, some samples also produced DTX1 or OA. The DTX1 content found in many *D. fortii* samples was considerably higher than that in *D. acuminata*. OA was detected as a minor toxin in some samples collected from Noheji and Yakumo. Several *D. fortii* samples from Noheji and Yakumo did not have any of the monitored toxins. PTX2 seco-acid was detected in *D. fortii* collected in Hakata. The PTX2 content (236.0 pg/cell) of *D. fortii* collected in Akita represents the highest value ever reported (Table 1) [23].

### 2.3. Other Dinophysis Species

**Figure 4:**
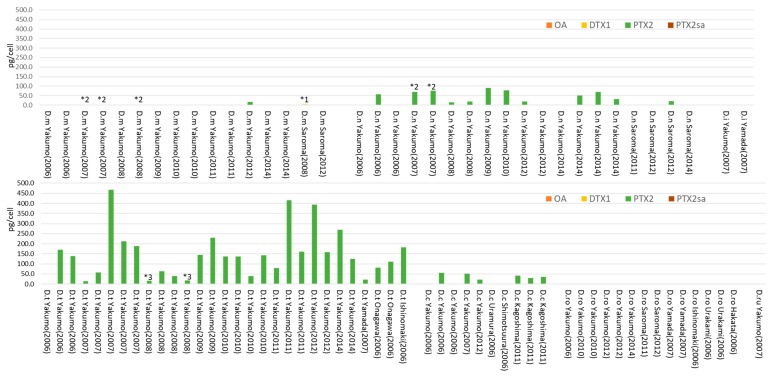
Toxin contents and the profiles of other *Dinophysis* species in Japan. D.m; *D. mitra*, D.n; *D. norvegica*, D.i; *D. infundibulus*, D.t; *D. tripos*, D.c; *D. caudata*, D.ro; *D. rotundata*, D.ru; *D. rudgei*. * 1 Trace levels of OA were detected. * 2 Trace levels of DTX1 were detected. * 3 Trace levels of PTX2 were detected. The toxin contents, profiles, analyzed cell numbers, and detection limits for negative values are also given in Table S1.

PTX2 was the only toxin detected in many other *Dinophysis* species collected and analyzed as part of this study, including *D. norvegica*, *D. tripos*, and *D. caudata*. Trace levels of DTX1 or OA were observed in some of these samples (Figure 4, Table S1). PTX2 was detected for the first time in *D. mitra* from Yakumo (2012) by LC/MS when using selected ion monitoring (SIM) in positive ion mode. The highest PTX2 content per cell of a *D. tripos* found in this study was 467.4 pg/cell, which represents the highest value ever reported (Table 1). It was also interesting that some of the other *Dinophysis* species collected and identified (e.g., *D. rudgei*) did not produce any of the monitored toxins, which aligns with the observations from *D. acuminata* and *D. fortii* isolates. Some *D. mitra* and *D. rotundata* samples, showed trace levels of DTX1 or OA. *D. norvegica* collected in Yakumo also contained a low level of DTX1. 

## 3. Discussion

In this study, the toxin content and profiles of *Dinophysis* species collected around the Japanese coastline were determined. Novel findings include the detection of DTX1 in *D. norvegica* and PTX2 in *D. caudata*. OA or DTX1 have been reported in *D. norvegica* from coastal waters in other countries [19,24]. Detection of PTX2 in pooled picked cells of *D. caudata* in Japan reported for the first time, however, it has been detected in Spanish and Chinese strains of this species [25,29,32]. Detection of PTX2 in pooled picked cells of *D. tripos* and *D. mitra* is also a novel observation, although PTX2 has been detected in cultures of *D. tripos* isolated from Japan [14]. Due to the very low concentration of PTX2 observed in the *D. mitra* samples, its presence was not confirmed by LC/MS/MS fragment ions, and further confirmation will be required.

LC-MS analyses of picked cells of *Phalacroma rotundatum* (*D. rotundata*) showed small amounts of the same toxins (OA, DTXs, PTXs) present in the co-occurring *Dinophysis* species or no toxins at all. These observations led to the hypothesis that the heterotrophic *P. rotundatum* is not a de novo toxin-producer, but a vector of DSP toxins taken up from its tintinnid prey. [36]. The small amount of DTX1 observed in *D. rotundata*, and heterotroph that feedss on tintinids collected and analyzed as part of our study might be derived from DTX1 produced by other co-occurring *Dinophysis* species present in the area.

This study determined that the most dominant toxin produced by *Dinophysis* species in Japan is PTX2, except for some samples of *D. acuminata*, *D. fortii*, *D. rotundata*, and *D. mitra* that produced only OA or DTX1. PTX11, which had been detected in *D. acuta* from Spain and New Zealand [8,9,39], was not detected in any *Dinophysis* samples from Japan. This indicates that, in Japan, there is little risk of bivalves being contaminated with PTX11. It was interesting that there were non-toxic *Dinophysis* samples even within the same species. This demonstrates the difficulty in predicting contamination of bivalves with DSTs or PTXs by monitoring *Dinophysis* cell densities. Monitoring of DSTs and PTXs in plankton net samples or SPATT devices [40] by LC/MS/MS methods could be useful for early warnings of bivalve contamination with these toxins.

In Japan, the LC/MS/MS method [38] for OA/DTX analogues has been introduced as the official testing method for DSTs since 2015 [41]. In terms of early warning of bivalve contamination with DSTs, *D. fortii* could be regarded as the most important *Dinophysis* species to monitor because the DTX1 contents of *D. fortii* were relatively higher than those found in other *Dinophysis* species. It is interesting that sampling sites that showed DTX1-containing *D. fortii* coincided well with the historical human poisoning cases of DSP [42,43,44]. When the percentages of DTX1 and OA from *D. fortii* samples in Japan were compared, those of DTX1 are greater. This result is consistent with the fact that the dominant OA analogue in Japanese bivalves is DTX1 and 7-*O*-acyl-DTX1 [45,46]. It is also noteworthy that *D. acuminata* from Takada produces a relatively high amount of OA. Although there have been no human DSP cases in this area, attention should be payed to prevent future cases when high cell densities of *D. acuminata* were observed in this area.

Monitoring of DSP in Japanese bivalves has historically been performed using the mouse bioassay (MBA). This methodology was implemented as the official testing method for the DSP monitoring program established in the 1980s [47]. Although the presence of *Dinophysis* had been confirmed in southeastern regions (Tokai, Kinki, Shikoku, Sanyo, Sanin, Kyusyu regions), in Japan, there had been a few MBA positive cases in bivalves from those regions. This could be explained by the results of this study showing the dominant toxin in *Dinophysis* collected in the southwestern regions (Hakata, Shimonoseki, Kagoshima, Hiroshima) is PTX2, which is then converted to a MBA non-toxic PTX2SA in many bivalve species (Pacific oyster, manila clam, etc.). The exception to this is Japanese scallops, *Patinopecten yessoensis* (*Mizuhopecten yessoensis*), cultured in northeastern Japan [37,48]. However, as *D. acuminata* collected in Takada produces a sufficiently high amounts of OA, there is a risk of human poisoning by DSTs when there is high cell densities of *D. acuminata* in this region. Therefore, continuous monitoring of DSP in bivalves around the coastline of Japan is necessary.

## 4. Materials and Methods

### 4.1. Chemicals

Okadaic acid (OA) and dinophysistoxin-1 (DTX1), pectenotoxin (PTX-1, 2, 6), and yessotoxin (YTX) were produced by the Japanese reference material project [49]. PTX-11 was isolated from *D. acuta* collected in New Zealand [8]. Methanol and acetonitrile, and formic acid of LC/MS grade were purchased from Kanto chemical co., Tokyo, Japan And ammonium formate of analytical grade was purchased from Nacalai tesque co., Tokyo, Japan. Distilled water was prepared by milli-Q Reference (Merck Millipore, Burlington, MA, USA).

### 4.2. Sampling Locations and Dinophysis Sample Preparation

**Figure 5:**
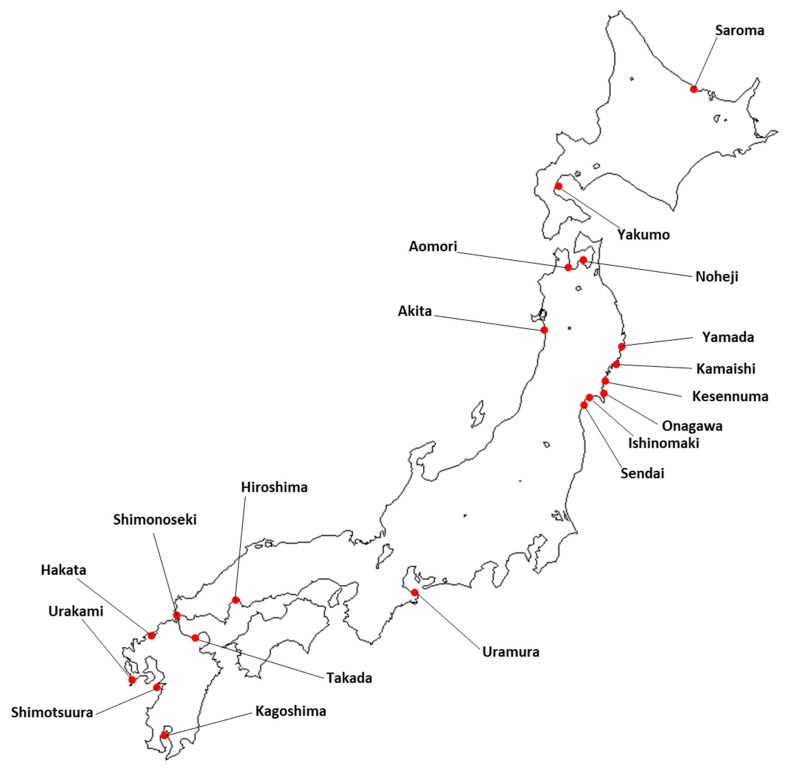
*Dinophysis* sampling locations around Japan.

Seawater samples were collected from various locations around the Japanese coastline (Figure 5). Using a light microscope, 50 individual cells of *Dinophysis* species identified in the seawater samples were carefully selected using a glass capillary to exclude non-targeted microorganisms. The cells were identified by their morphological characteristics. Isolated *Dinophysis* cells that had been taxonomically identified were combined in a single vessel filled with filtered seawater and stored frozen until extraction. Detailed information on the sampling is shown in Table S1.

### 4.3. Extraction

Toxins were extracted from cells of *Dinophysis* species by solid phase extraction (SPE) (Sep pak C18 plus, Waters co., Milford, MA, USA) as reported in previous studies [6,19,23]. Toxin extracts were dissolved in 200 µL of methanol for LC/MS/MS analysis.

### 4.4. LC/MS/MS and LC/MS Analysis

LC/MS/MS analysis was carried out according to a previous method [23]. The LC/MS/MS system was an Agilent 1100 series of high performance liquid chromatograph (HPLC) (Agilent technologies, Lexington, MA, USA) coupled with a 3200 Qtrap triple quadrupole MS/MS system (Sciex, Framingham, MA, USA). Separations were performed on Quicksilver cartridge columns (50 mm × 2.1 mm i.d) packed with 3 µm Hypersil-BDS-C8 (Keystone Scientific, Bellefonte, PA, USA) and maintained at 20 °C. Eluent A was water and B was acetonitrile–water (95:5), both containing 2 mM ammonium formate and 50 mM formic acid [50,51]. A linear gradient elution from 20% to 100% B was performed over 10 min and then held at 100% B for 15 min, followed by re-equilibration with 20% B (13 min). The flow rate was 0.2 mL/min and the injection volume was 10 µL. MRM LC/MS/MS analysis for the targeted toxins were carried out using the following ions; [M − H]^−^ (OA, DTX1, 7-*O*-palmitoyl-DTX1, DTX2, PTX6, PTX2sa, YTX, 45OHYTX) and [M + HCOOH − H]^−^ (PTX1, PTX2, PTX11) as the target parent ions in Q1 and particular fragment ions of each toxin in Q3 as reported in a previous study [40]. SIM LC/MS analysis for toxins were carried out using the [M + NH_4_]^+^ (OA, DTX1, DTX2, PTX1, PTX2, PTX6, PTX11) as the target ions in Q1.

## Figures and Tables

**Table 1:** Reported toxin content and profiles in pooled picked cell isolates of *Dinophysis* field specimens.

Species	pg/cell				Location	Analysis Method	Reference
	OA	DTX1	DTX2	PTX2			
***Dinophysis acuminata***	1.6	-	-	-	Le Havre, France	HPLC-FLD	[19]
	Trace	-	-	-	Tokyo Bay, Japan	HPLC-FLD	[19]
	9.1	-	-	-	Gullmar, Sweden	HPLC-FLD	[20]
	9.9–21.7	-	-	-	Galicia, Spain	HPLC-FLD	[21]
	-	-	-	180.0	Bahia Inglesa, Chile	LC/MS/MS	[22]
	-	0.3–0.7	-	10.7–22.4	Abashiri, Japan	LC/MS/MS	[23]
	-	ND–0.7	-	25.9–50.2	Yakumo, Japan	LC/MS/MS	[23]
	ND–0.8	-	-	0.9–8.7	Flødevigen Bay, Noway	LC/MS/MS	[24]
	3.7	-	-	-	Bueu, Spain	LC/MS/MS	[25]
***Dinophysis fortii***	-	13.0–191.5	-	42.5	Mutsu Bay, Japan	HPLC-FLD	[19]
	23.0	-	-	-	Inland Sea, Japan	HPLC-FLD	[19]
	ND–57.7	ND–16.0	-	-	Ofunato, Japan	HPLC-FLD	[26]
	-	8.4–10.9	-	51.4–63.8	Yakumo, Japan	LC/MS/MS	[23]
***Dinophysis acuta***	9.4	-	-	-	Vigo, Spain	HPLC-FLD	[19]
	4.0	4.2	-	-	Sogndal, Norway	HPLC-FLD	[19]
	-	6.6	-	-	Gullmar, Sweden	HPLC-FLD	[20]
	58.0	-	78.0	-	Ireland	HPLC-FLD	[27]
	6.3–33.1	-	1.0–22.0	-	Galicia, Spain	HPLC-FLD	[21]
	85.0	-	77.0	14.0	Glandore, Ireland	LC/MS/MS	[28]
	-	-	-	29.1–32.3	Galicia, Spain	LC/MS/MS	[29]
	0.7–9.4	-	0.9–6.6	0.3–3.3	Pontevedra, Spain	LC/MS	[30]
	1.0–8.5	-	-	0.2–3.3	Flødevigen Bay, Noway	LC/MS/MS	[24]
	2.9	-	1.9	1.5	Bueu, Spain	LC/MS/MS	[25]
***Dinophysis caudata***	0.7	-	-	-	Galicia, Spain	HPLC-FLD	[21]
	7.9–56.5	ND–53.9	-	-	Sapian, Phillipines	HPLC-FLD	[31]
	-	-	-	100.0–127.4	Galicia, Spain	LC/MS/MS	[29]
	0.6	-	2.8	5.0	Moana, Spain	LC/MS/MS	[25]
	-	-	-	2.0–14.5	Day Bay, China	LC/MS/MS	[32]
***Dinophysis infundibulus***	-	-	-	14.8	Yakumo, Japan	LC/MS/MS	[23]
***Dinophysis miles***	5.7–20.9	ND–10.7	-	-	Sapian, Phillipines	HPLC-FLD	[31]
***Dinophysis mitra***	-	10.0	-	-	Mutsu Bay, Japan	HPLC-FLD	[19]
	-	-	-	-	Yakumo, Japan	LC/MS/MS	[23]
***Dinophysis norvegica***	-	14.0	-	-	Sogndal, Norway	HPLC-FLD	[19]
	-	-	-	50.8–67.4	Yakumo, Japan	LC/MS/MS	[23]
	ND–0.2	-	-	0.3–1.7	Flødevigen Bay, Noway	LC/MS/MS	[24]
***Dinophysis ovum***	7.1	-	-	-	Vigo, Spain	LC/MS/MS	[33]
***Dinophysis rotundata***	ND–0.4	-	ND–0.5	ND–0.3	Bueu, Spain	LC/MS/MS	[34]
	-	101.0	-	-	Mutsu Bay, Japan	HPLC-FLD	[19]
	-	-	-	-	Yakumo, Japan	LC/MS/MS	[23]
	-	-	-	0.8	Flødevigen Bay, Noway	LC/MS/MS	[24]
***Dinophysis sacculus***	16.5	-	-	-	Le Croisic, France	HPLC-FLD	[35]
	14.0	-	-	-	Morgat, France	HPLC-FLD	[35]
	29.6	-	-	-	Kervel, France	HPLC-FLD	[35]
	12.9	-	-	-	Pont-Aven, France	HPLC-FLD	[35]
***Dinophysis skagii***	-	-	-	-	Bueu, Spain	LC/MS/MS	[25]
***Dinophysis tripos***	-	36.0	-	-	Kesennuma, Japan	HPLC-FLD	[19]
	-	-	-	-	Yakumo, Japan	LC/MS/MS	[23]

## Supplementary Materials

The following are available online at http://www.mdpi.com/2072-6651/10/11/457/s1, Table S1: Toxin profiles of *Dinophysis* species collected from around the coast of Japan.
